# Supplementary material for: Selectivity of Terpyridine Platinum Anticancer Drugs for G-quadruplex DNA
Source: Molecules. 2019 Jan 23;24(3):404. doi: 10.3390/molecules24030404 (PMC6385020; doi:10.3390/molecules24030404)
Supplement: Supplementary file 1 [file molecules-24-00404-s001.pdf]

Supporting information

Selectivity of Terpyridine platinum anti-cancer drugs for G-quadruplex DNA

Elodie Morel [1,2,†], Claire Beauvineau [1,2,†], Delphine Naud-Martin[1,2], Corinne Landras-Guetta[1,2], Daniela Verga[1,2], Deepanjan Ghosh[1,2], Sylvain Achelle[1,2,3], Florence Mahuteau-Betzer[1,2], Sophie Bombard[1,2*] and Marie-Paule Teulade-Fichou[1,2*]

1 Institut Curie, PSL Research University, CNRS-UMR 9187, INSERM U1196, F-91405, Orsay, France.

2 Université Paris Sud, Université Paris-Saclay, CNRS-UMR 9187, INSERM U1196, F-91405 Orsay, France

3 Univ Rennes, CNRS, ISCR-UMR 6226, F-35000 Rennes, France

* Correspondence marie-paule.teulade-fichou@curie.fr (M-P.T-F) and sophie.bombard@curie.fr (S.B); Tel.: 33169863086 and 33169863189

† These authors contributed equally to this work.

A

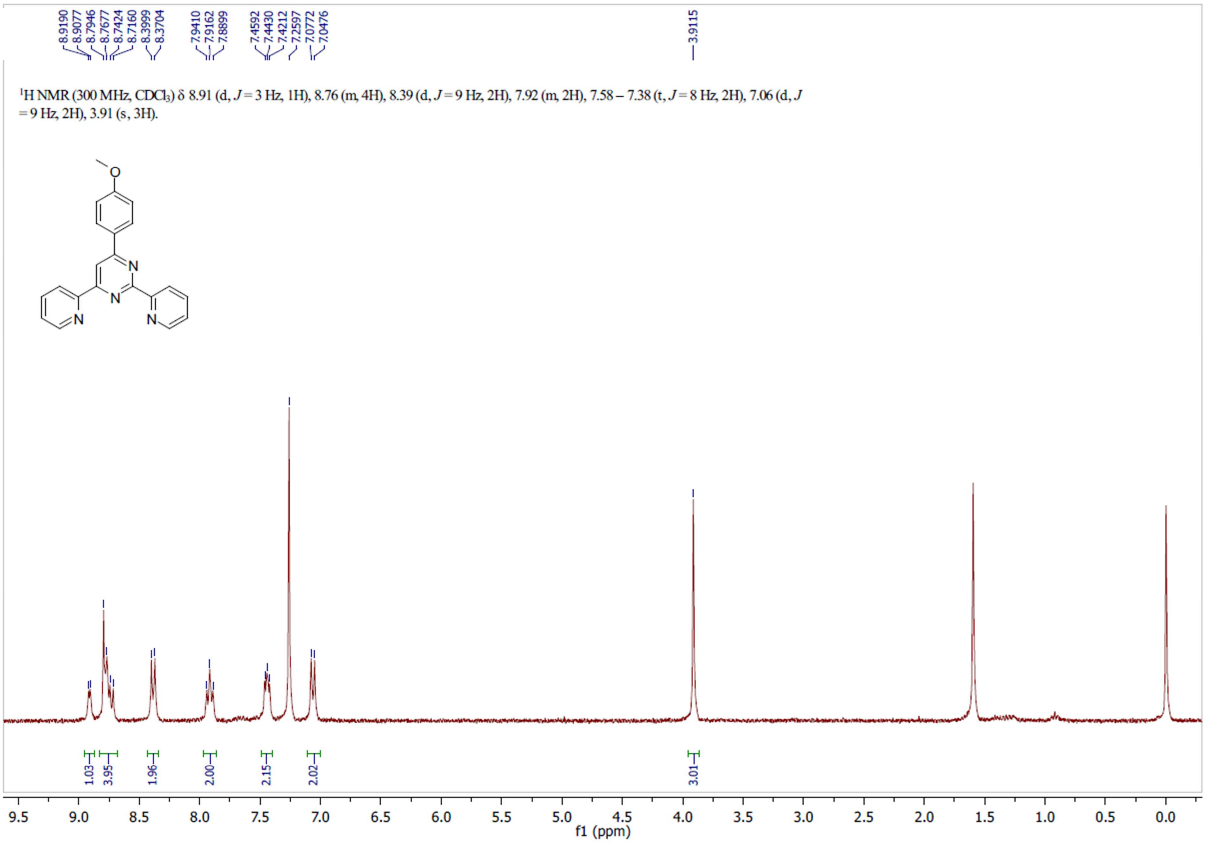

^{1}H NMR (300 MHz, CDCl$_3$) δ 8.91 (d, J = 3 Hz, 1H), 8.76 (m, 4H), 8.39 (d, J = 9 Hz, 2H), 7.92 (m, 2H), 7.58 – 7.38 (t, J = 8 Hz, 2H), 7.06 (d, J = 9 Hz, 2H), 3.91 (s, 3H).
f1 (ppm)

B

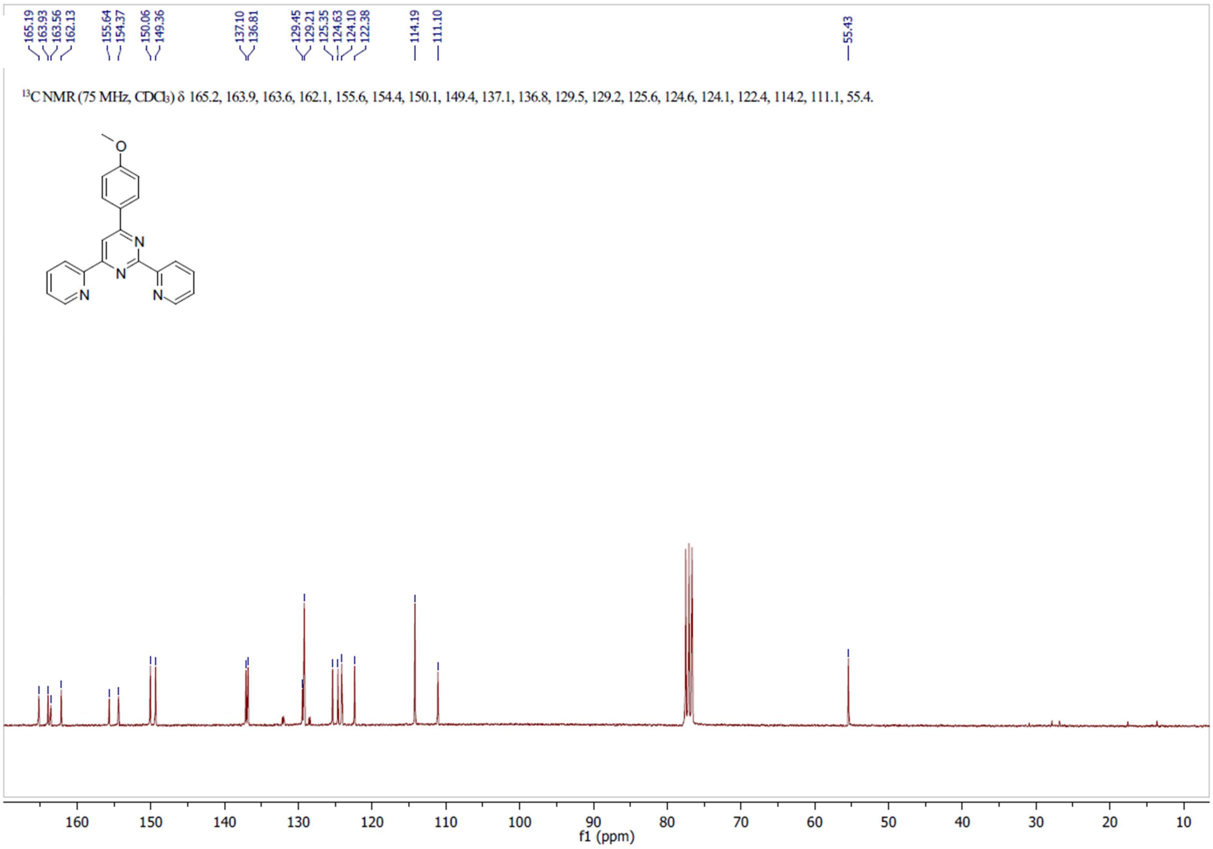

^{13}C NMR (75 MHz, CDCl$_3$) δ 165.2, 163.9, 163.6, 162.1, 155.6, 154.4, 150.1, 149.4, 137.1, 136.8, 129.5, 129.2, 125.6, 124.6, 124.1, 122.4, 114.2, 111.1, 55.4.
f1 (ppm)

C

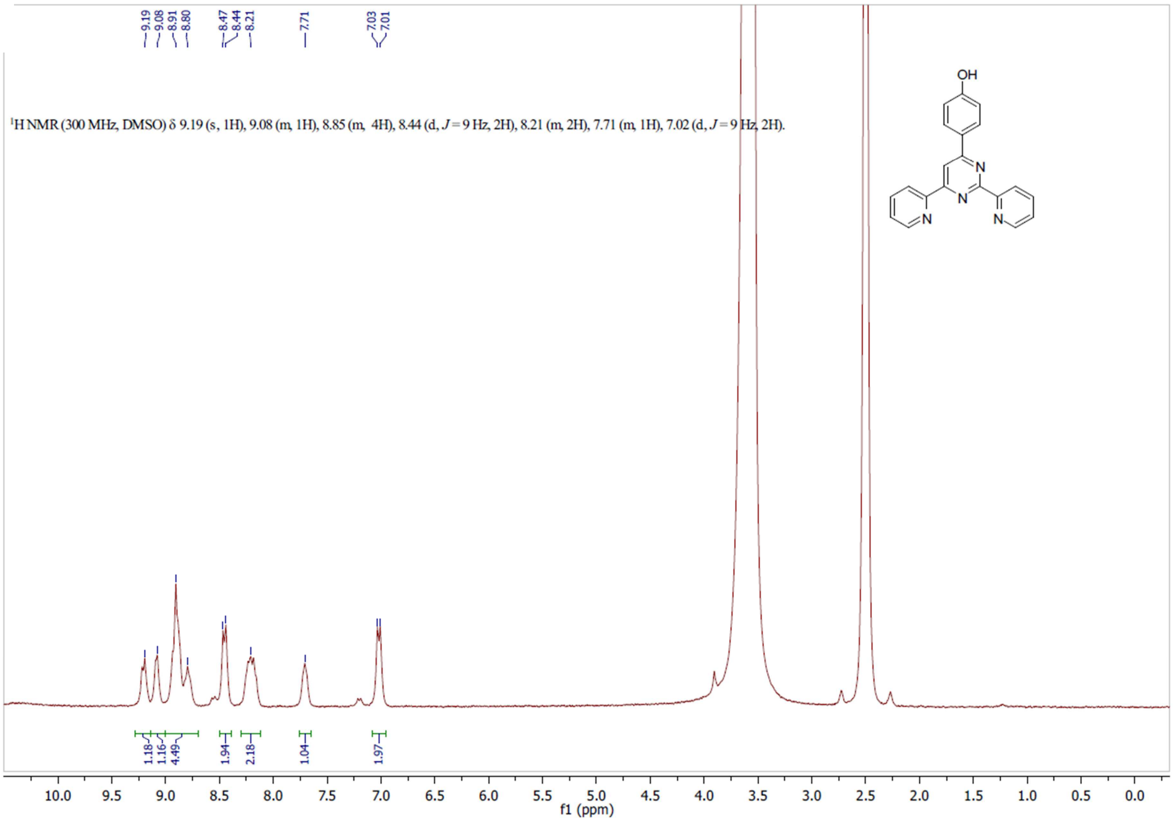
9.19
9.08
8.91
8.80
8.47
8.44
8.21
7.71
7.03
7.01
^{1}H NMR (300 MHz, DMSO) δ 9.19 (s, 1H), 9.08 (m, 1H), 8.85 (m, 4H), 8.44 (d, J = 9 Hz, 2H), 8.21 (m, 2H), 7.71 (m, 1H), 7.02 (d, J = 9 Hz, 2H).
OH
N
N
N
N
1.18
1.16
4.49
1.94
2.18
1.04
1.97
10.0 9.5 9.0 8.5 8.0 7.5 7.0 6.5 6.0 5.5 5.0 4.5 4.0 3.5 3.0 2.5 2.0 1.5 1.0 0.5 0.0
f1 (ppm)

D

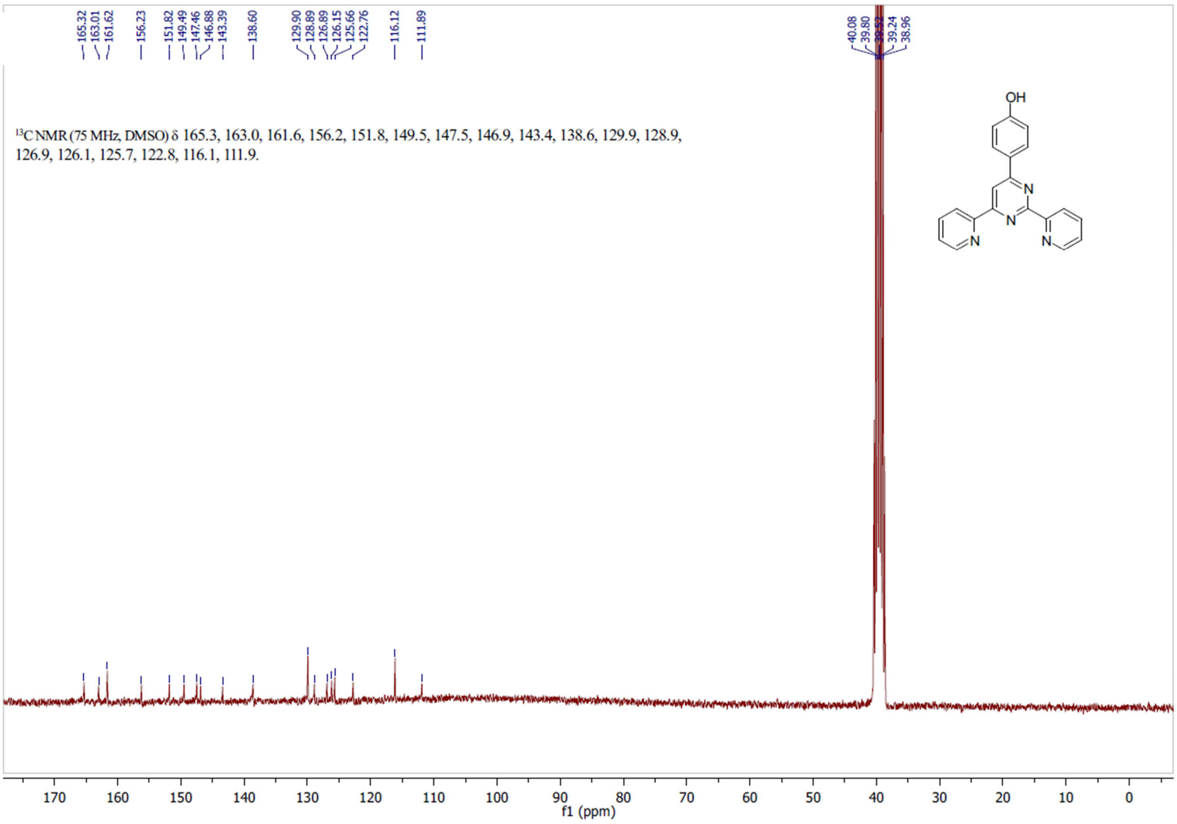
165.32
163.01
161.62
156.23
151.82
149.49
147.46
146.88
143.39
138.60
129.90
128.89
126.89
126.15
125.66
122.76
116.12
111.89
40.08
39.80
39.24
38.96
^{13}C NMR (75 MHz, DMSO) δ 165.3, 163.0, 161.6, 156.2, 151.8, 149.5, 147.5, 146.9, 143.4, 138.6, 129.9, 128.9, 126.9, 126.1, 125.7, 122.8, 116.1, 111.9.
OH
N
N
N
N
170 160 150 140 130 120 110 100 90 80 70 60 50 40 30 20 10 0
f1 (ppm)

E

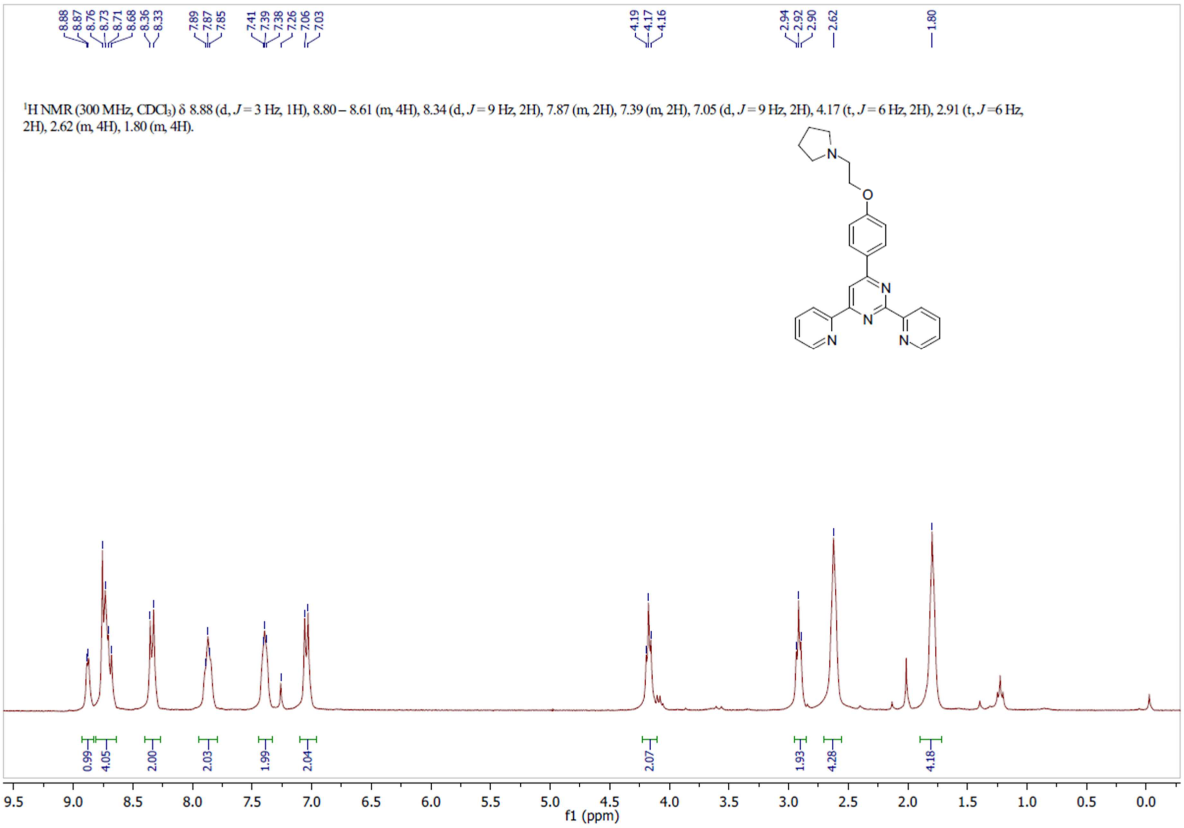

¹H NMR (300 MHz, CDCl₃) δ 8.88 (d, J = 3 Hz, 1H), 8.80 – 8.61 (m, 4H), 8.34 (d, J = 9 Hz, 2H), 7.87 (m, 2H), 7.39 (m, 2H), 7.05 (d, J = 9 Hz, 2H), 4.17 (t, J = 6 Hz, 2H), 2.91 (t, J =6 Hz, 2H), 2.62 (m, 4H), 1.80 (m, 4H).
8.88 8.87 8.76 8.73 8.71 8.68 8.36 8.33 7.89 7.87 7.85 7.41 7.39 7.38 7.26 7.06 7.03 4.19 4.17 4.16 2.94 2.92 2.90 2.62 1.80
0.99 4.05 2.00 2.03 1.99 2.04 2.07 1.93 4.28 4.18
f1 (ppm)

F

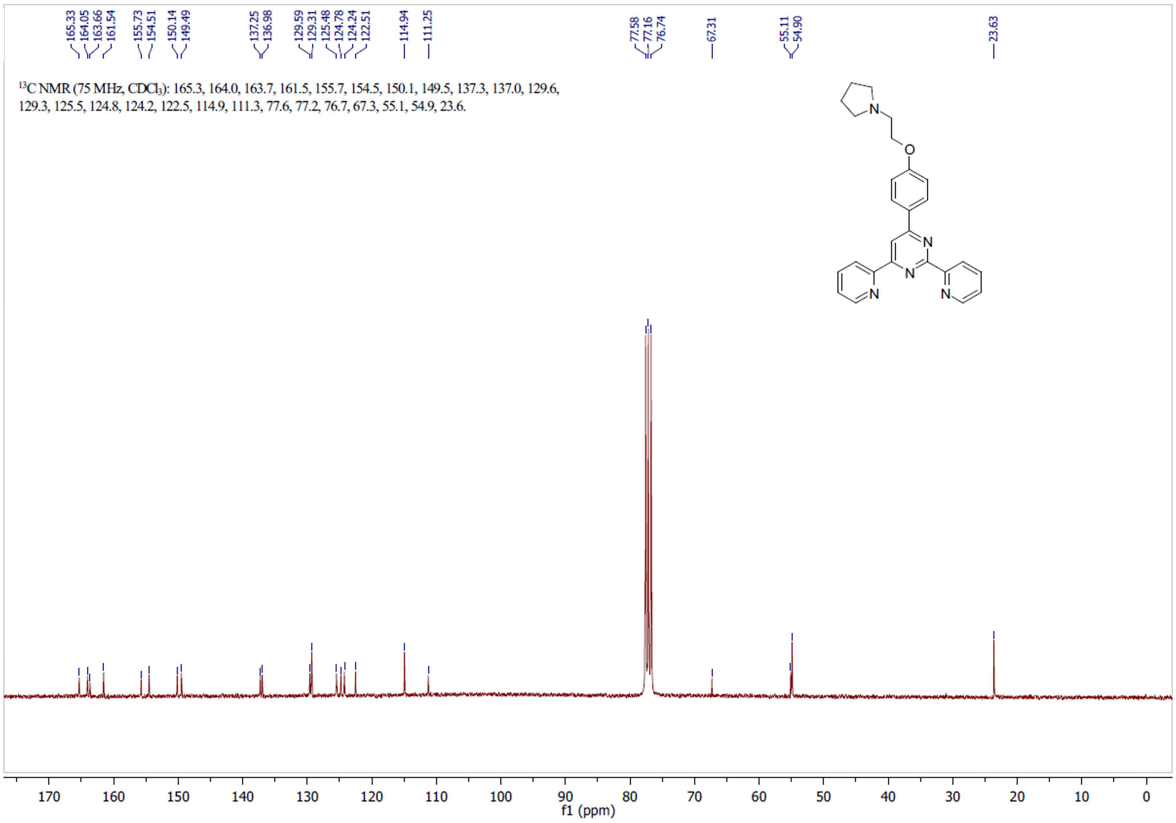

¹³C NMR (75 MHz, CDCl₃): 165.3, 164.0, 163.7, 161.5, 155.7, 154.5, 150.1, 149.5, 137.3, 137.0, 129.6, 129.3, 125.5, 124.8, 124.2, 122.5, 114.9, 111.3, 77.6, 77.2, 76.7, 67.3, 55.1, 54.9, 23.6.
165.33 164.05 163.66 161.54 155.73 154.51 150.14 149.49 137.25 136.98 129.59 129.31 125.48 124.78 124.24 122.51 114.94 111.25 77.58 77.16 76.74 67.31 55.11 54.90 23.63
f1 (ppm)

G

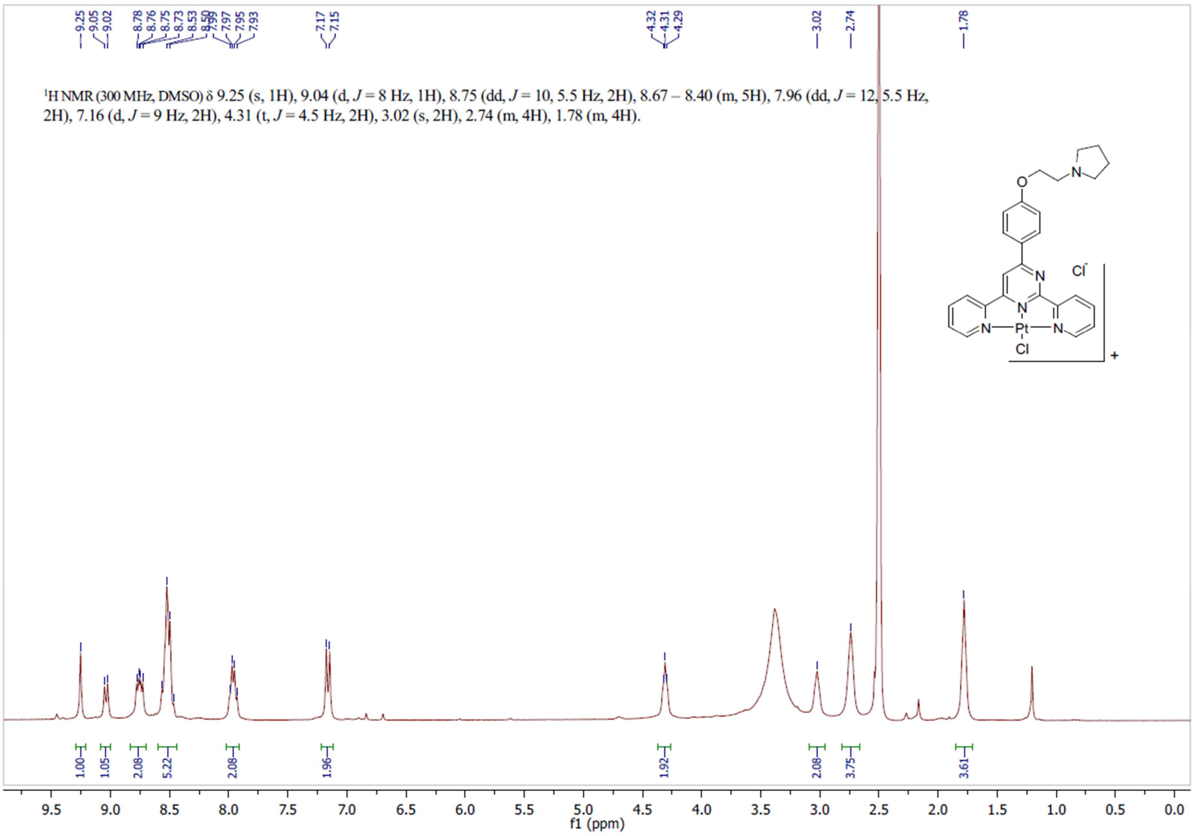

¹H NMR (300 MHz, DMSO) δ 9.25 (s, 1H), 9.04 (d, J = 8 Hz, 1H), 8.75 (dd, J = 10, 5.5 Hz, 2H), 8.67 – 8.40 (m, 5H), 7.96 (dd, J = 12, 5.5 Hz, 2H), 7.16 (d, J = 9 Hz, 2H), 4.31 (t, J = 4.5 Hz, 2H), 3.02 (s, 2H), 2.74 (m, 4H), 1.78 (m, 4H).
f1 (ppm)

H

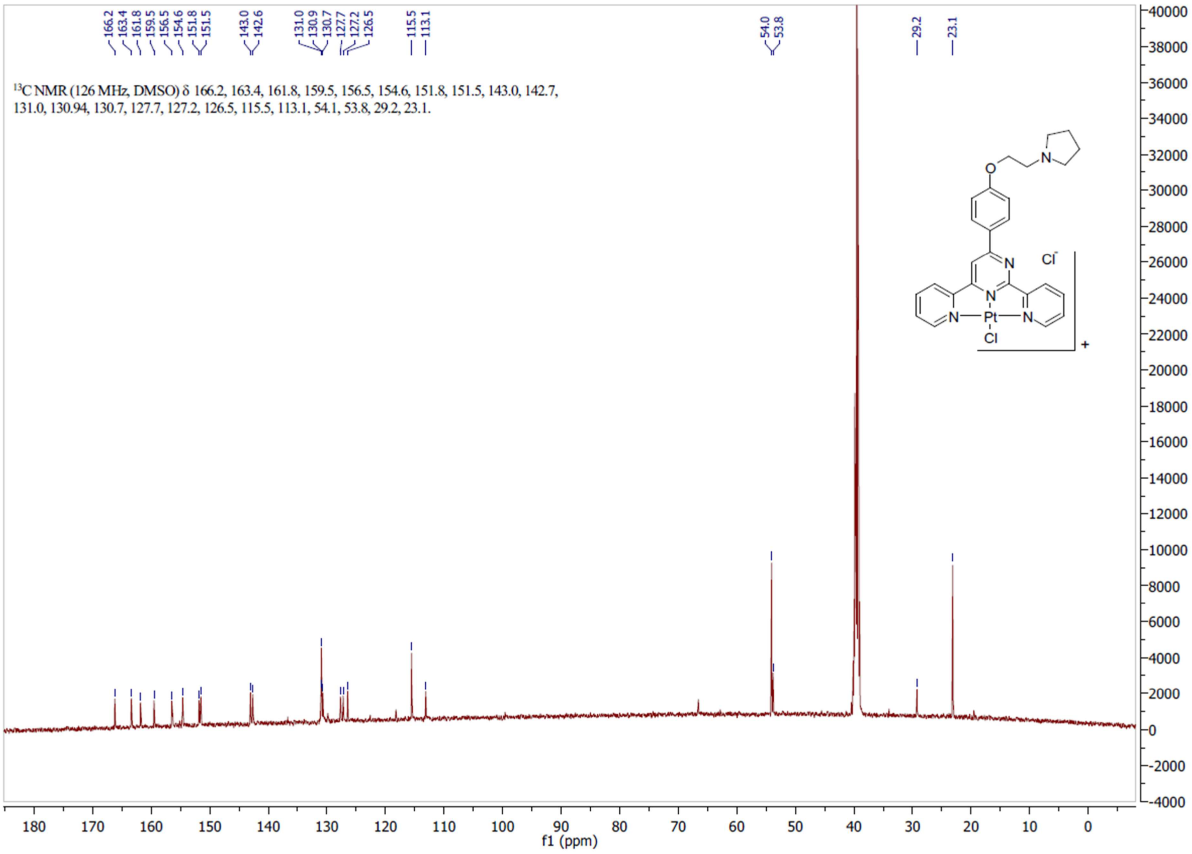

¹³C NMR (126 MHz, DMSO) δ 166.2, 163.4, 161.8, 159.5, 156.5, 154.6, 151.8, 151.5, 143.0, 142.7, 131.0, 130.94, 130.7, 127.7, 127.2, 126.5, 115.5, 113.1, 54.1, 53.8, 29.2, 23.1.
f1 (ppm)

I

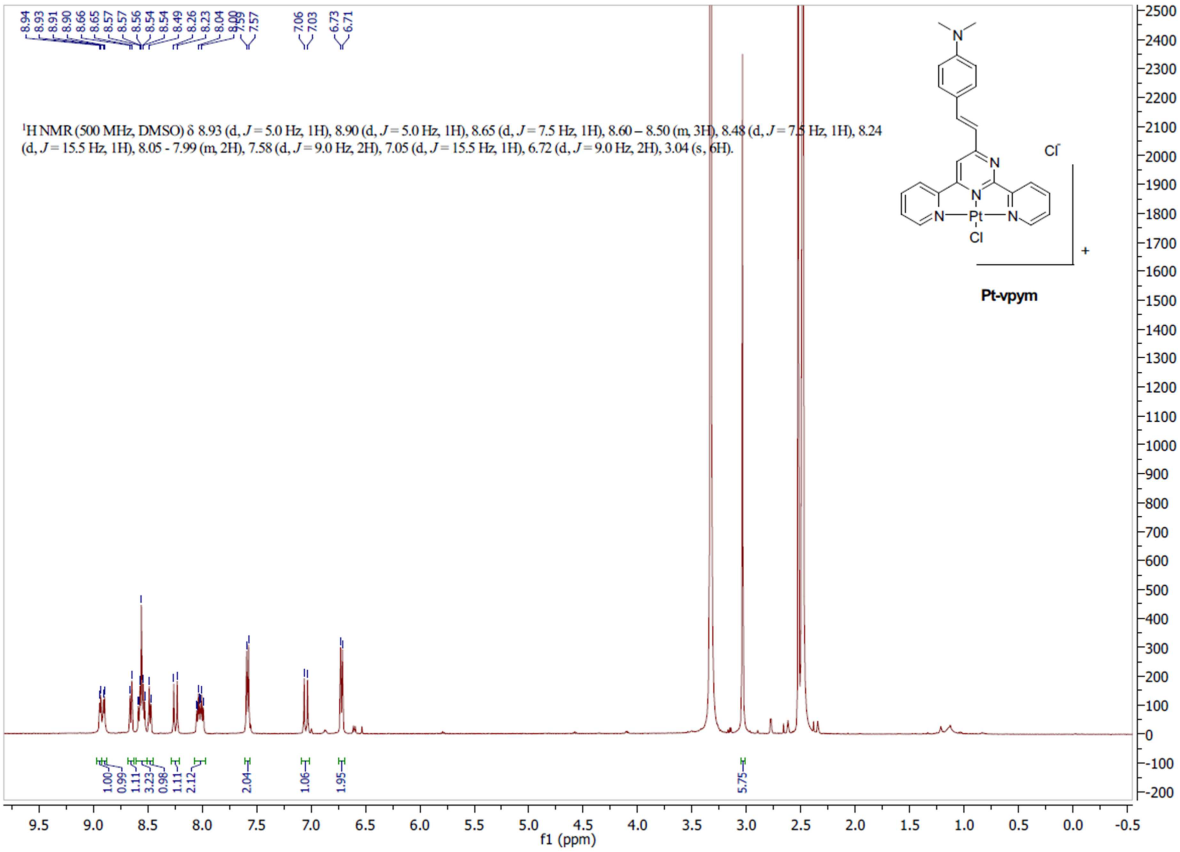
^{1}H NMR (500 MHz, DMSO) δ 8.93 (d, J = 5.0 Hz, 1H), 8.90 (d, J = 5.0 Hz, 1H), 8.65 (d, J = 7.5 Hz, 1H), 8.60 – 8.50 (m, 3H), 8.48 (d, J = 7.5 Hz, 1H), 8.24 (d, J = 15.5 Hz, 1H), 8.05 - 7.99 (m, 2H), 7.58 (d, J = 9.0 Hz, 2H), 7.05 (d, J = 15.5 Hz, 1H), 6.72 (d, J = 9.0 Hz, 2H), 3.04 (s, 6H).
Pt-vpym
f1 (ppm)

J

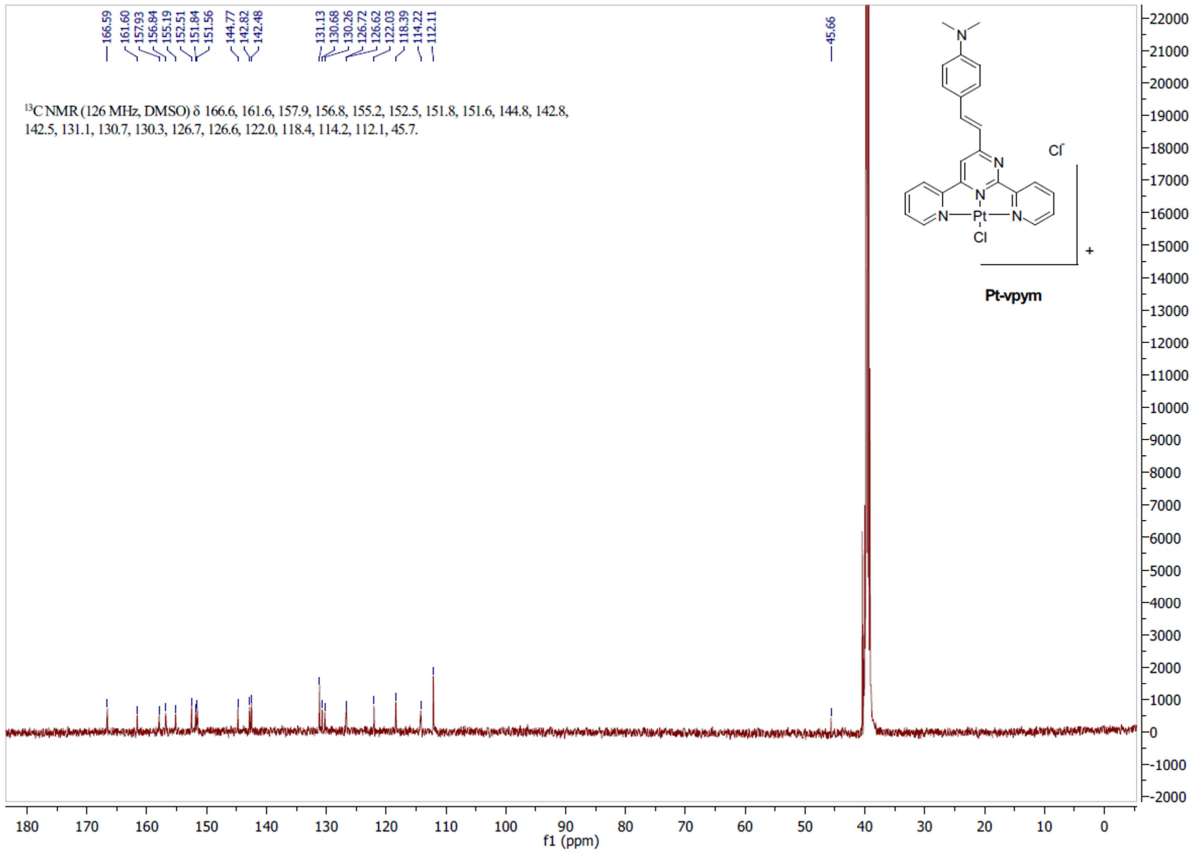
^{13}C NMR (126 MHz, DMSO) δ 166.6, 161.6, 157.9, 156.8, 155.2, 152.5, 151.8, 151.6, 144.8, 142.8, 142.5, 131.1, 130.7, 130.3, 126.7, 126.6, 122.0, 118.4, 114.2, 112.1, 45.7.
Pt-vpym
f1 (ppm)

K

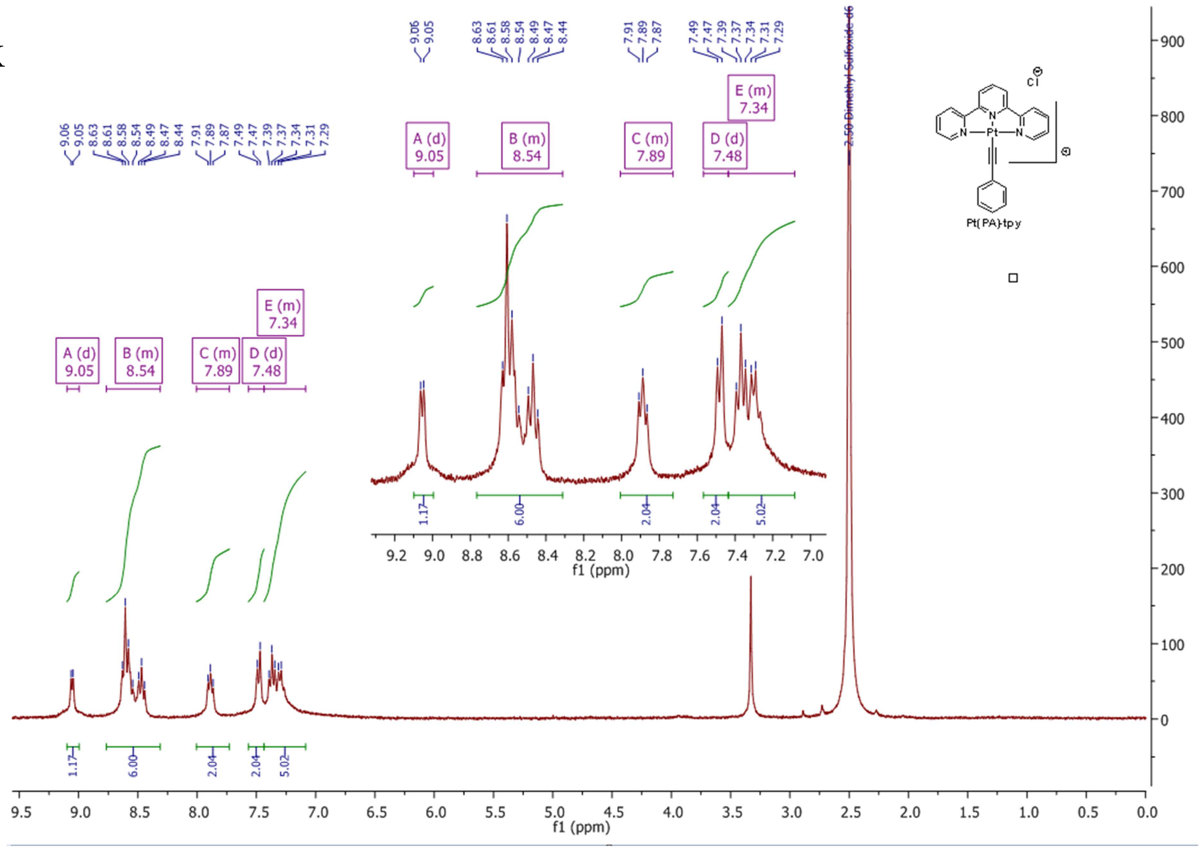

Figure S1: ^{1}H NMR and ^{13}C spectra, respectively, of compound **2** (A, B), compound **3** (C, D), **cpym** (E, F), **Pt-cpym** (G, H), **Pt-vpym** (I, J) and ^{1}H NMR spectrum of **Pt(PA)-tpy** (K).

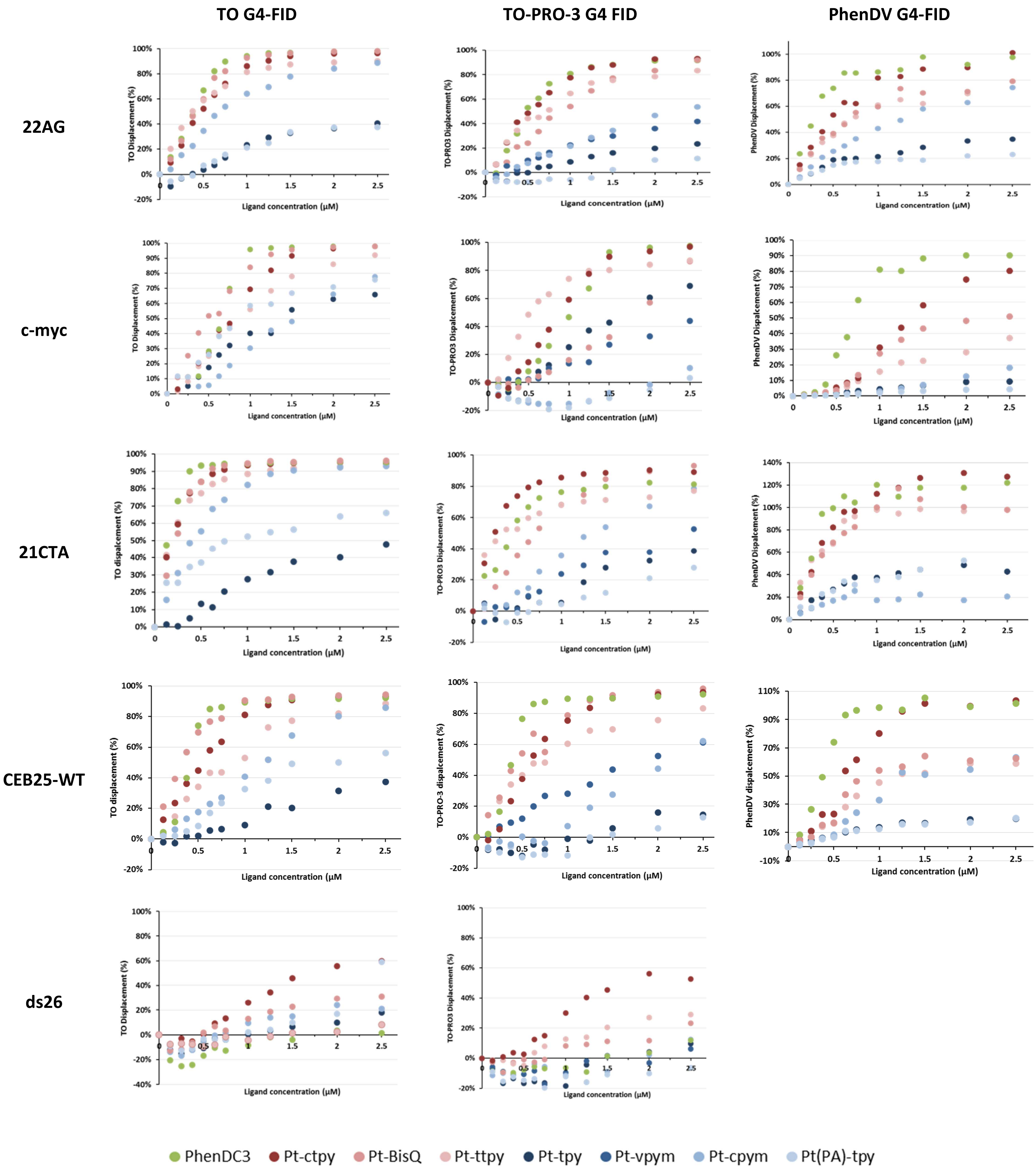

Figure S2. Fluorescence Intercalator Displacement (FID) assays, for compounds **Pt-BisQ**, **Pt-ctpy**, **Pt-ttpy**, **Pt-cpym**, **Pt-vpym**, **Pt(PA)-tpy** and **Pt-tpy** with G4 DNA (22AG, c-myc, 21CTA and 25CEBWT, 0,25 µM) in presence of Thiazole Orange (TO), TO-PRO-3 (2 equivalents), and PhenDV (1.5 equivalents). Affinities of ligands are expressed by probe displacement as indicated in material and methods. Buffer used is K^+100 (10 mM lithium cacodylate, 100 mM KCl and 1 % DMSO, pH = 7.3).

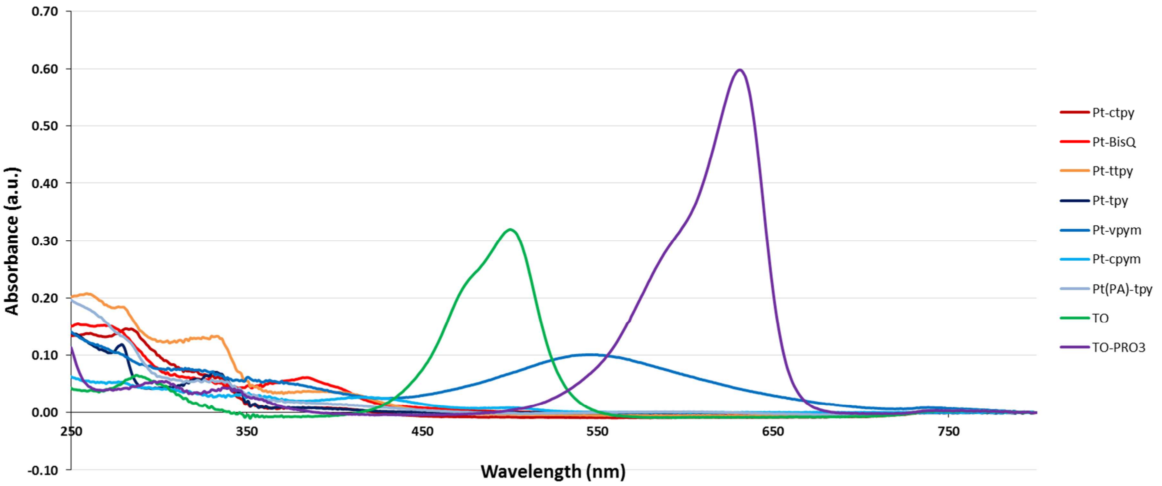

Figure S3. UV-Vis spectra of **Pt-complexes,** TO and TO-PRO-3 at 5 µM in water.

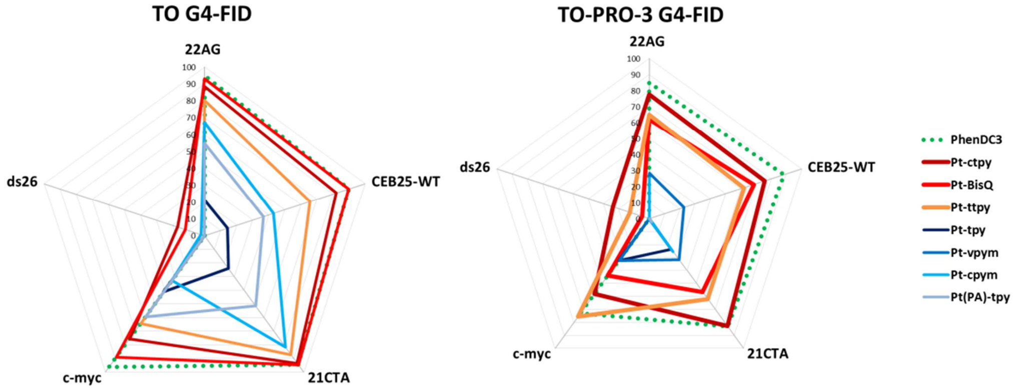

Figure S4: % of probe displacement at 1µM ligand (**Pt-BisQ**, **Pt-ctpy**, **Pt-ttpy**, **Pt-cpym**, **Pt-vpym**, **Pt(PA)-tpy** and **Pt-tpy** and PhenDC3 used as control) from G4-FID assay. Experiments are performed on the G-quadruplex structure of 22AG, c-myc, 21CTA, CEB25WT (as shown in Figure 1A) and on duplex ds26 DNA (0,25 µM), with thiazole orange (TO) (2 eq. for G4-DNA & 3eq. for ds26), TO-PRO-3 (2 eq. for G4-DNA & 3eq. for ds26) in K^+100 buffer (100 mM KCl and 10 mM lithium cacodylate, pH = 7.3).

PLATINATION OF G-QUADRUPLEX DNA

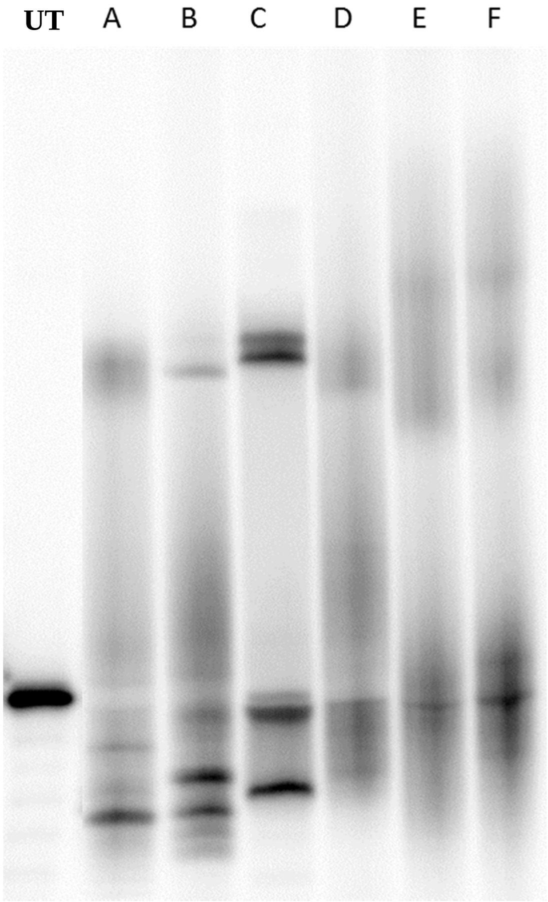

Figure S5. Denaturating gel electrophoresis (15 % acrylamide) of platination reactions induced by 2 equivalents of compounds **Pt-ttpy** (A), **Pt-tpy** (B), Pt-BisQ (C), **Pt-ctpy** (D), **Pt-cpym** (E), and **Pt-vpym** (F) on c-myc (100 µM) in the presence of K^+100 buffer after 18h incubation at 32°C. Lane labelled with UT corresponds to untreated c-myc.

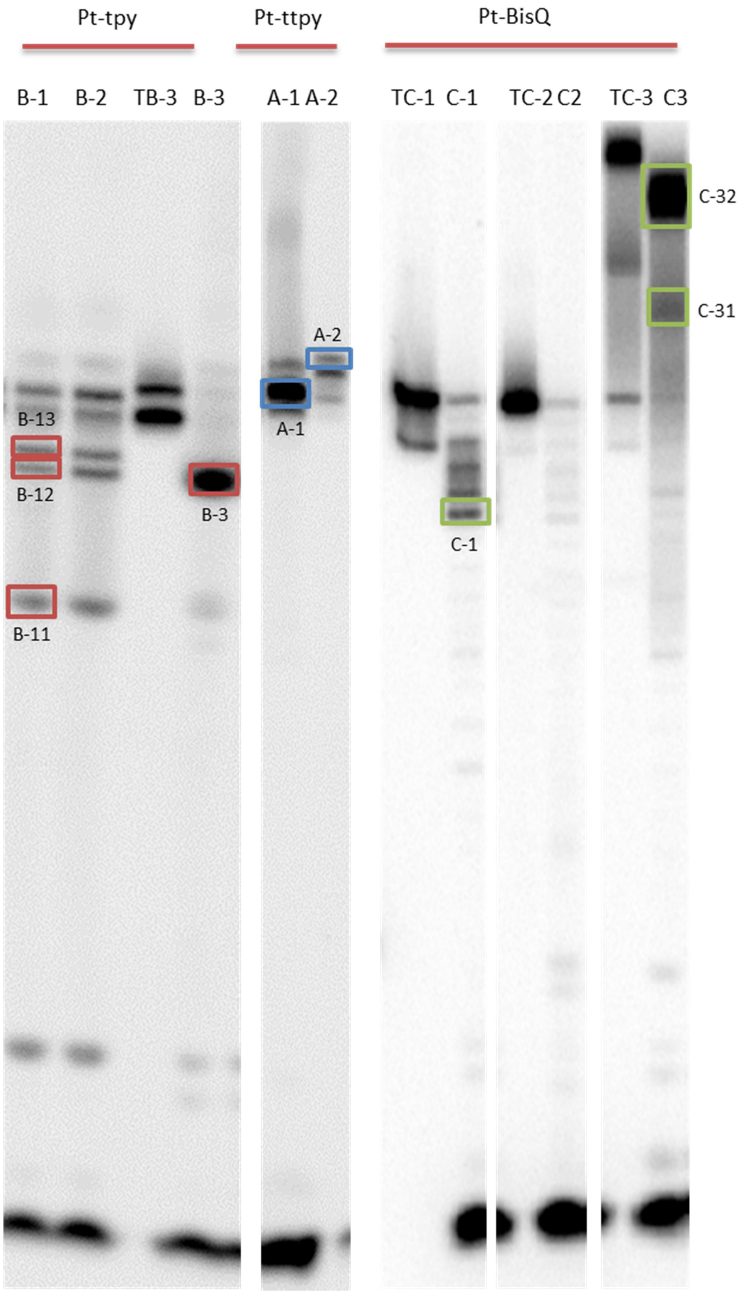

Figure S6. Denaturating gel electrophoresis (20% acrylamide) of 3′-exonuclease digestion of bands obtained by platination of c-myc with **Pt-ttpy**, **Pt-tpy** and **Pt-BisQ** from Figure 4. Lane labelled with T corresponds to the non-digested bands.

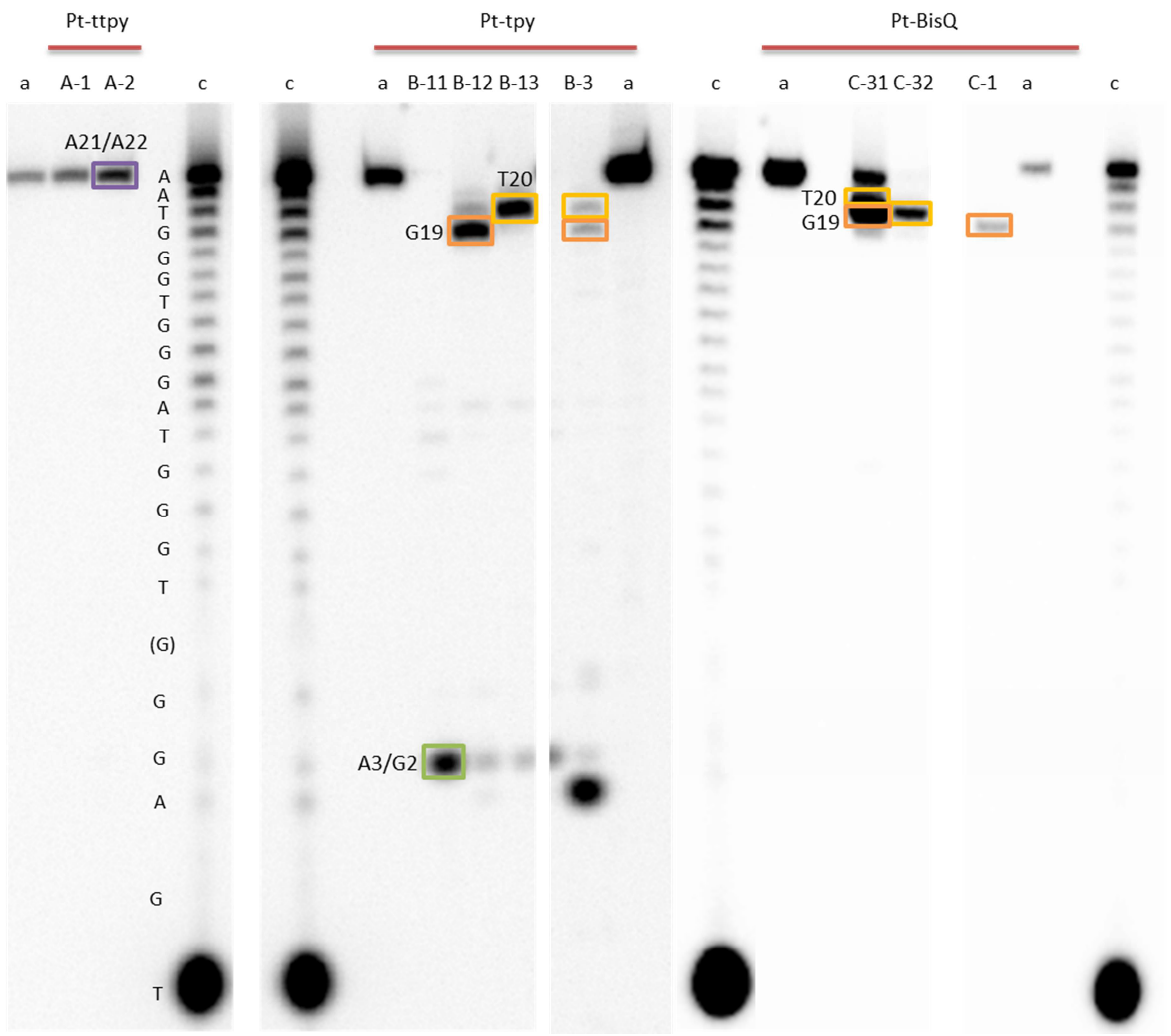

Figure S7. Denaturing gel electrophoresis (20% acrylamide) of the fragments of digestion from Figure S6 after deplatination using NaCN 0.2M at 32 °C for 18 hours. Lane c corresponds to the partial digestion of non-platinated DNA c-myc (0.008 unit enzyme/μM), and it gives the reference scale for the migration of the deplatinated digested fragments. Lane a corresponds to the non-digested oligonucleotide c-myc. The letter and number indicate the platinum binding site.

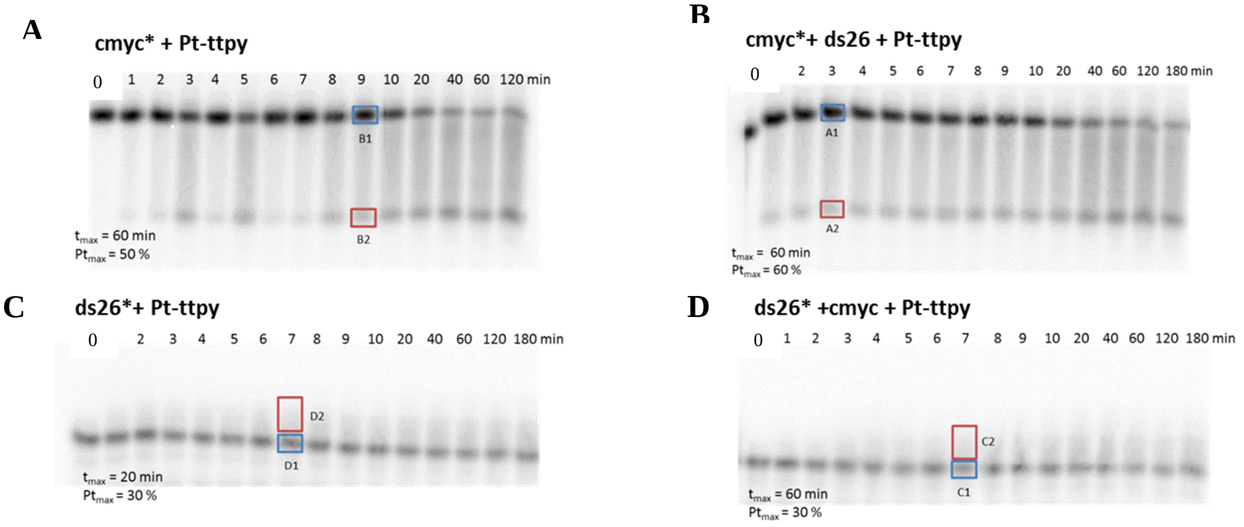

Figure S8. Kinetics of formation of platinated adduct of ^{32}P radiolabeled c-myc* (0.2 µM) (A) and cold duplex DNA ds26 (10 µM) (B) or of ^{32}P radiolabeled ds26*(10 µM) (C) and cold c-myc (0.2 µM) (D) in the presence of **Pt-ttpy** (5 µM) in K^+10 buffer at 25°C followed by denaturing acrylamide gel electrophoresis.

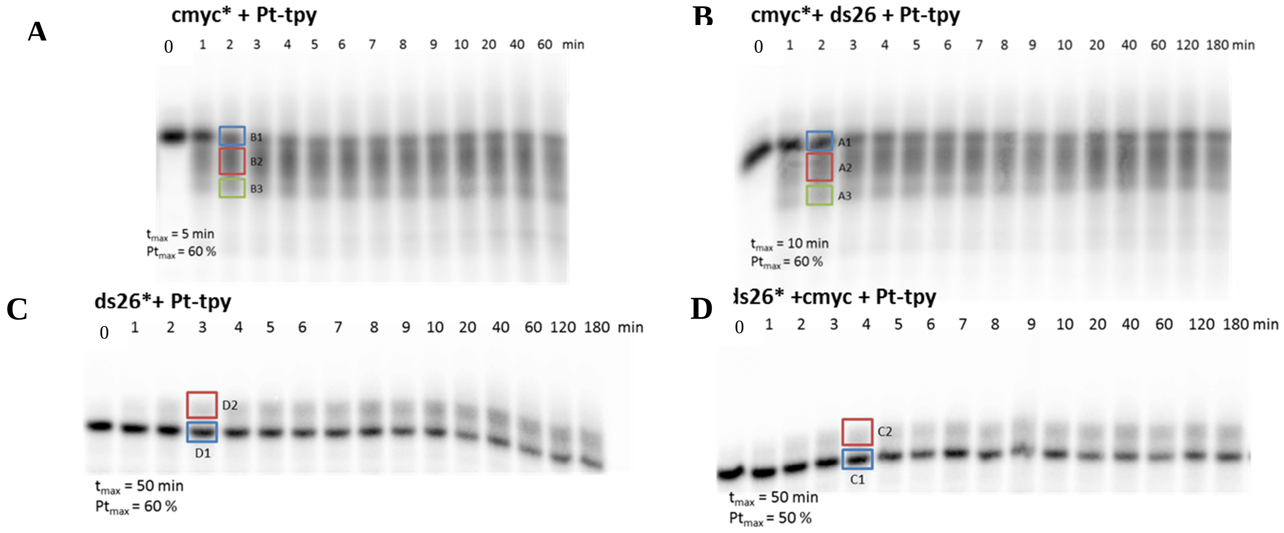

Figure S9. Kinetics of formation of platinated adduct of ^{32}P radiolabeled c-myc* (0.2 µM) (A) and cold duplex DNA ds26 (10 µM) (B) or of ^{32}P radiolabeled ds26* (10 µM) (C) and cold c-myc (0.2 µM) (D) in the presence of **Pt-tpy** (5 µM) in K^+10 buffer at 25°C followed by denaturing acrylamide gel electrophoresis.

Table S1: ΔTm (°C) of F-21-T, F-c-myc-T, F-21CTA-T and F-CEB25-WT-T in the presence of **Pt-ctpy**, **Pt-BisQ**, **Pt-ttpy**, **Pt-tpy**, **Pt-vpym**, **Pt-cpym**, **Pt(PA)-tpy**, and PhenDC3.

Ligand	ΔTm (°C)							
	F-21-T		F-c-myc-T		F-21CTA-T		F-CEB25-WT-T	
	0 μM ds26	10 μM ds26	0 μM ds26	10 μM ds26	0 μM ds26	10 μM ds26	0 μM ds26	10 μM ds26
Pt-ctpy	16.7±1.1	7.4±1.0	13.0±2.1	6.4±1.3	14.2±0.9	4.7±0.6	17.0±1.9	3.9±0.5
Pt-BisQ	20.6±2.0	8.9±1.0	10.3±1.8	3.7±0.4	10.6±1.3	0.4±0.5	15.6±4.1	3.9±0.6
Pt-ttpy	12.8±2.9	3.9±1.9	9.4±2.9	5.5±2.6	7.5±3.0	1.8±1.2	10.0±3.8	1.6±0.6
Pt-tpy	6.0±1.2	2.1±0.5	3.2±1.8	3.2±2.9	7.0±1.5	6.6±1.0	4.2±1.3	2.3±2.9
Pt-vpym	7.9±2.0	5.3±0.5	3.5±0.9	8.5±2.8	3.7±0.9	6.8±0.3	3.8±0.5	1.8±0.1
Pt-cpym	10.3±2.1	1.1±0.1	1.7±0.4	1.6±1.4	0.8±0.1	0.9±0.3	2.2±0.3	0.6±0.0
Pt(PA)-tpy	5.2±1.4	-1.0±0.4	0.4±0.5	0.4±0.7	0.3±0.3	-0.5±0.3	0.8±0.3	0.1±0.1
PhenDC3	24.3±1.4	24.4±1.0	21.3±1.2	21.7±0.7	18.4±1.0	14.6±1.2	22.0±1.3	19.8±1.7

Table S2: % of TO displacement at 1 μM of ligand (**Pt-ctpy**, **Pt-BisQ**, **Pt-ttpy**, **Pt-ypy**, **Pt-cpym**, **Pt(PA)-tpy**, and PhenDC3) on 22AG, C-myc, 21CTA, CEB-25WT and ds26.

Ligand	% of TO displacement at 1 μM of ligand				
	22AG	c-myc	21CTA	CEB25-WT	ds26
Pt-ctpy	88	76	93	82	17
Pt-BisQ	93	89	95	90	12
Pt-ttpy	80	64	87	66	nd
Pt-tpy	21	41	24	14	nd
Pt-cpym	67	33	82	43	2
Pt(PA)-tpy	55	59	52	37	nd
PhenDC3	95	96	95	90	nd

Table S3: % of TO-PRO-3 displacement at 1 μM of ligand (**Pt-ctpy**, **Pt-BisQ**, **Pt-ttpy**, **Pt-tpy**, **Pt-vpym**, **Pt-cpym**, **Pt(PA)-tpy**, and PhenDC3) on 22AG, C-myc, 21CTA, CEB-25WT and ds26.

Ligand	% of TOPRO3 displacement at 1 μM of ligand				
	22AG	c-myc	21CTA	CEB25-WT	ds26
Pt-ctpy	78	58	83	76	24
Pt-BisQ	62	44	57	69	5
Pt-ttpy	65	76	62	62	13
Pt-tpy	12	32	23	nd	nd
Pt-vpym	28	32	32	23	nd
Pt-cpym	25	nd	25	nd	nd
Pt(PA)-tpy	nd	nd	3	nd	nd
PhenDC3	85	73	83	88	nd

Table S4: % of PhenDV displacement at 1 μM of ligand (**Pt-ctpy**, **Pt-BisQ**, **Pt-ttpy**, **Pt-tpy**, **Pt-cpym**, **Pt(PA)-tpy**, and PhenDC3) on 22AG, C-myc, 21CTA and CEB25-WT.

Ligand	% of PhenDV displacement at 1 μM of ligand			
	22AG	c-myc	21CTA	CEB25-WT
Pt-ctpy	84	29	100	72
Pt-BisQ	64	20	94	45
Pt-ttpy	63	22	90	45
Pt-tpy	24	5	35	13
Pt-cpym	43	3	72	33
Pt(PA)-tpy	20	2	34	13
PhenDC3	92	85	100	99
